# Improving the Compliance with NICE Guidelines, for the Physical Health Monitoring of Young Persons, on Treatment for Attention Deficiet Hyperactivity Disorder (ADHD), in West of Glasgow Child and Adolescent Mental Health Service (CAMHS)

**DOI:** 10.1192/bjo.2025.10440

**Published:** 2025-06-20

**Authors:** Salomee Shakur

**Affiliations:** NHS Greater Glasgow and Clyde, Glasgow, United Kingdom

## Abstract

**Aims:** To improve the compliance with the NICE (National Institute for Health and Care Excellence) physical health monitoring guidelines, for young persons on medications, for ADHD, attending the West of Glasgow CAMHS, by at least 50%.

**Methods:** A quality improvement project was implemented to improve the compliance. As intervention staff education, multiple poster placement and introduction of the BP Percentile app was made within the team. The PDSA (Plan-Do-Study-Act) cycle was used to test the change in ideas.

**Results:** The repeat audit done 5 weeks after implementing the above interventions, resulted in a 19% increase in Blood Pressure recorded within 6 months along with 47% increase in them recorded as percentiles.

**Conclusion:** The results of the study showed marked improvement in the compliance in all aspects, especially in the recording of Blood Pressures in percentiles. The improvement was from 0% to 47% almost reaching the goal of 50%. Having said that there is still room for improvement! Future change of ideas include adding an EMIS template which automatically calculates the values in percentiles and re-auditing the cycle after the changes are implemented.

